# Supplementary material for: Influenza A Virus Infection Reactivates Human Endogenous Retroviruses Associated with Modulation of Antiviral Immunity
Source: Viruses. 2022 Jul 21;14(7):1591. doi: 10.3390/v14071591 (PMC9320126; doi:10.3390/v14071591)
Supplement: Supplementary file 1 [file viruses-14-01591-s001.zip › MotifAnalysis.zip!/MotifOutput_SC35M/knownResults.html]

MotifOutput/ - Homer Known Motif Enrichment Results


# Homer Known Motif Enrichment Results (MotifOutput/)

Homer *de novo* Motif Results  
Gene Ontology Enrichment Results  
Known Motif Enrichment Results (txt file)  
Total Target Sequences = 41, Total Background Sequences = 47106

|  |  |  |  |  |  |  |  |  |  |  |  |
| --- | --- | --- | --- | --- | --- | --- | --- | --- | --- | --- | --- |
| Rank | Motif | Name | P-value | log P-pvalue | q-value (Benjamini) | # Target Sequences with Motif | % of Targets Sequences with Motif | # Background Sequences with Motif | % of Background Sequences with Motif | Motif File | SVG |
| 1 | T C G A C T A G A G T C A G T C C G T A C G T A A C G T T A G C T C A G T A C G | NFY(CCAAT)/Promoter/Homer | 1e-5 | -1.287e+01 | 0.0011 | 13.0 | 31.71% | 3288.0 | 6.98% | motif file (matrix) | svg |
| 2 | A T G C A G T C G C T A C G A T C G T A G C A T G C T A C G A T C T A G C A T G T G A C G T C A | CArG(MADS)/PUER-Srf-ChIP-Seq(Sullivan\_et\_al.)/Homer | 1e-4 | -1.099e+01 | 0.0037 | 8.0 | 19.51% | 1331.2 | 2.83% | motif file (matrix) | svg |
| 3 | G C A T C G A T C T A G G C T A G C A T C G T A G A T C G C T A C G A T C G A T C T A G C G A T C G T A C G A T G A T C | DMRT1(DM)/Testis-DMRT1-ChIP-Seq(GSE64892)/Homer | 1e-4 | -1.064e+01 | 0.0037 | 7.0 | 17.07% | 1004.4 | 2.13% | motif file (matrix) | svg |
| 4 | G A C T C G A T C A T G G C T A G C A T C G T A A G T C G C T A G C A T C G A T T A C G G C A T C G T A C A G T G A T C | DMRT6(DM)/Testis-DMRT6-ChIP-Seq(GSE60440)/Homer | 1e-4 | -9.355e+00 | 0.0095 | 6.0 | 14.63% | 843.9 | 1.79% | motif file (matrix) | svg |
| 5 | A T G C G A C T A C G T C T A G A C G T A C G T A C G T C T G A G A T C G C T A A G C T C G T A | Foxa2(Forkhead)/Liver-Foxa2-ChIP-Seq(GSE25694)/Homer | 1e-3 | -8.206e+00 | 0.0240 | 10.0 | 24.39% | 3122.2 | 6.63% | motif file (matrix) | svg |
| 6 | A G C T A G T C A G T C A C G T C T A G A C G T A C G T A C G T C G T A A G T C G A T C C G T A | FOXP1(Forkhead)/H9-FOXP1-ChIP-Seq(GSE31006)/Homer | 1e-3 | -7.433e+00 | 0.0434 | 7.0 | 17.07% | 1691.5 | 3.59% | motif file (matrix) | svg |
| 7 | C T A G T A C G G A C T T G C A T G C A C G A T T A C G C T G A T C G A C T G A | Hoxa10(Homeobox)/ChickenMSG-Hoxa10.Flag-ChIP-Seq(GSE86088)/Homer | 1e-2 | -6.445e+00 | 0.0999 | 7.0 | 17.07% | 2004.7 | 4.26% | motif file (matrix) | svg |
| 8 | T C A G C T A G A T G C G A C T T G C A C G T A A G C T A C G T C T G A T A C G | En1(Homeobox)/SUM149-EN1-ChIP-Seq(GSE120957)/Homer | 1e-2 | -6.079e+00 | 0.1259 | 16.0 | 39.02% | 8945.9 | 19.00% | motif file (matrix) | svg |
| 9 | G C T A T C G A C G T A C T A G A G C T G T C A G T C A C G T A A G T C C G T A | FOXA1(Forkhead)/MCF7-FOXA1-ChIP-Seq(GSE26831)/Homer | 1e-2 | -6.039e+00 | 0.1259 | 10.0 | 24.39% | 4131.4 | 8.78% | motif file (matrix) | svg |
| 10 | G C T A T C G A C G T A C T A G A G C T G T C A G T C A C G T A A G T C C G T A | FOXA1(Forkhead)/LNCAP-FOXA1-ChIP-Seq(GSE27824)/Homer | 1e-2 | -5.832e+00 | 0.1290 | 11.0 | 26.83% | 4995.8 | 10.61% | motif file (matrix) | svg |
| 11 | T C G A A C T G A C T G C G T A C G T A T C G A A G T C C T G A A T C G G T A C G C A T C A T G | ETS:E-box(ETS,bHLH)/HPC7-Scl-ChIP-Seq(GSE22178)/Homer | 1e-2 | -5.706e+00 | 0.1330 | 3.0 | 7.32% | 343.0 | 0.73% | motif file (matrix) | svg |
| 12 | C G T A G C T A C G A T C T A G A C G T G T C A C G T A C G T A A G T C C G T A T G C A T A C G | FoxL2(Forkhead)/Ovary-FoxL2-ChIP-Seq(GSE60858)/Homer | 1e-2 | -5.672e+00 | 0.1330 | 9.0 | 21.95% | 3623.7 | 7.70% | motif file (matrix) | svg |
| 13 | C G A T C T A G A C G T G T C A C G T A C G T A A G T C C G T A | Foxo3(Forkhead)/U2OS-Foxo3-ChIP-Seq(E-MTAB-2701)/Homer | 1e-2 | -5.655e+00 | 0.1330 | 8.0 | 19.51% | 2948.9 | 6.26% | motif file (matrix) | svg |
| 14 | T A C G T A G C G C T A C G A T C T A G A C G T C A G T C A G T G C T A A G T C G T C A G C A T | FOXK2(Forkhead)/U2OS-FOXK2-ChIP-Seq(E-MTAB-2204)/Homer | 1e-2 | -5.282e+00 | 0.1597 | 7.0 | 17.07% | 2469.1 | 5.24% | motif file (matrix) | svg |
| 15 | A T C G G A C T C G A T C G T A C G T A C A G T G A T C G A T C G A T C A G C T | Otx2(Homeobox)/EpiLC-Otx2-ChIP-Seq(GSE56098)/Homer | 1e-2 | -4.659e+00 | 0.2779 | 10.0 | 24.39% | 5017.9 | 10.66% | motif file (matrix) | svg |
